# Interstitial lung disease associated with Equine Infectious Anemia Virus infection in horses

**DOI:** 10.1186/1297-9716-44-113

**Published:** 2013-12-01

**Authors:** Pompei Bolfa, Marie Nolf, Jean-Luc Cadoré, Cornel Catoi, Fabienne Archer, Christine Dolmazon, Jean-François Mornex, Caroline Leroux

**Affiliations:** 1 UMR754, Retrovirus and Comparative Pathology, INRA, Lyon, France; 2 Université Lyon 1, Lyon, France; 3 Université de Lyon, Lyon, France; 4 Hôpital Louis-Pradel, Hospices Civils de Lyon, Lyon, France; 5 Pathology Department, Faculty of Veterinary Medicine, University of Agricultural Sciences and Veterinary Medicine, 400372 Cluj-Napoca, Romania; 6 Equine department, VetAgro Sup, Campus vétérinaire de Lyon, France

## Abstract

EIA (Equine Infectious Anemia) is a blood-borne disease primarily transmitted by haematophagous insects or needle punctures. Other routes of transmission have been poorly explored. We evaluated the potential of EIAV (Equine Infectious Anemia Virus) to induce pulmonary lesions in naturally infected equids. Lungs from 77 EIAV seropositive horses have been collected in Romania and France. Three types of lesions have been scored on paraffin-embedded lungs: lymphocyte infiltration, bronchiolar inflammation, and thickness of the alveolar septa. Expression of the p26 EIAV capsid (CA) protein has been evaluated by immunostaining. Compared to EIAV-negative horses, 52% of the EIAV-positive horses displayed a mild inflammation around the bronchioles, 22% had a moderate inflammation with inflammatory cells inside the wall and epithelial bronchiolar hyperplasia and 6.5% had a moderate to severe inflammation, with destruction of the bronchiolar epithelium and accumulation of smooth muscle cells within the pulmonary parenchyma. Changes in the thickness of the alveolar septa were also present. Expression of EIAV capsid has been evidenced in macrophages, endothelial as well as in alveolar and bronchiolar epithelial cells, as determined by their morphology and localization. To summarize, we found lesions of interstitial lung disease similar to that observed during other lentiviral infections such as FIV in cats, SRLV in sheep and goats or HIV in children. The presence of EIAV capsid in lung epithelial cells suggests that EIAV might be responsible for the broncho-interstitial damages observed.

## Introduction

Equine Infectious Anemia Virus (EIAV) is a retrovirus belonging to the lentivirus genus, which infects equids worldwide (reviewed in [1]). Persistent infection is characterized by recurring viremic febrile episodes. Clinical signs of EIA (Equine Infectious Anemia), the EIAV-induced disease, are variable although infected equids often experience acute febrile episodes with concomitant thrombocytopenia and wasting syndrome followed by a chronic stage characterized by recurring disease episodes. In most cases, these febrile episodes eventually cease and despite remaining persistently infected the animals enter a prolonged phase with no overt clinical signs (reviewed in [1]). These inapparent carriers are potential reservoirs for transmission to naive equids. Despite a sanitary policy including culling of the seropositive animals and serological survey of all the in-contact horses, the disease still spreads among horses.

EIAV infection is endemic in horse populations throughout the world [2] including recent outbreaks in Belgium, Germany, Greece, France, Italy, Romania and UK (source OIE reports). Romania had to deal with more than 10 000 infected horses over the 2009–2012 period [3]. Relatively little is known about the origins, the viral characterization and the pandemic spread of the viruses [4,5].

In experimentally-infected equids, active viral replication has been detected in various tissues such as spleen, liver, lung, lymph nodes or bone marrow (reviewed in [1]), and viral tropism for mononuclear phagocytes is widely accepted. As for other human and animal lentiviruses, the cells of the monocyte/macrophage lineage represent the main targets of EIAV *in vivo*, supporting active viral replication during clinical and subclinical infection [6]. EIAV viral RNAs have been also detected in vascular endothelial cells in experimentally infected horses during the acute phase [7]. We recently demonstrated that EIAV is involved in the development of oxidative stress in horses [8].

We hypothesized that, as demonstrated with other lentiviruses, EIAV may induced specific lung lesions. We systematically characterized lung lesions from paraffin embedded sections collected from 93 Romanian and French horses. We stained the lung for the expression of EIAV p26 capsid protein. In this study we described for the first time an interstitial lung disease in EIAV infected horses as well as infection of lung epithelial cells.

## Materials and methods

### Animal source and tissue specimens

Lungs from 93 horses have been collected for this study. Seventy nine lungs (# 1 to 79) came from EIAV-positive (EIAV^POS^) horses, mean age 10.65 years (± 5.43) and from 14 EIAV-negative (EIAV^NEG^) horses (# 80 to 93), mean age 9.73 years (± 5.14). Among the EIAV^POS^ horses, 77 (31 females and 46 males, mean age 10.58 years (± 5.47)) have been collected in Romania (-rom) from October 2010 to May 2011. They were serologically positive for EIAV as confirmed by agar gel immunodiffusion test (AGID) or Coggins test (EIA Kit No. 119, Pasteur Institute) performed by regional reference laboratories across the country in the context of the wide range National survey of EIAV infection among farm horses. Positive animals have been put to death according to the national plan for eradication of EIA in Romania (Order No. 52/09.06.2010, ANSVSA Bucharest, Romania). At that time, none of these animals displayed clinical signs of EIA nor pulmonary disease as assessed by a rapid examination; importantly no biological parameters (body temperature or blood cell counts) have been collected. Lungs from 2 EIAV^POS^ French horses (78-fr, 10 year-old female and 79-fr, 16-year old male) have been collected on April 2005 (kindly provided by Dr Stephan Zientara, Veterinary school, Maisons-Alfort, France).

Fourteen EIAV^NEG^ horses, 7 males and 7 females (mean age 9.92 (± 4.94)), have been collected in France (-fr) and Romania (-rom). Five of these came from Romanian slaughterhouses (3 females and 2 males, mean age 9.00 years (± 6.06)) and nine French horses (mean age 10.14 years (± 5.01)) have been put to death at the Veterinary School of Lyon for colic (*n* = 6), septic shock (*n* = 2) or inguinal abscess (*n* = 1). While the EIAV serological status has been confirmed for the Romanian animals, none of the French horses have been serologically tested for EIAV. France does not have a wide range National survey of EIAV infection and the AGID is only performed for suspected animals presenting with clinical signs relevant to EIA or on “in-contact” animals with an already confirmed EIAV^POS^ animal. But importantly, none of the French EIAV^NEG^ horses had symptoms associated with EIAV and the prevalence in France being low, we considered these animals EIAV^NEG^ and associated them to the Romanian EIAV^NEG^ horses to constitute the control group.

### Pathology

Lungs were sampled from the mid dorsal part of the caudal lobe. Samples were fixed in 10% neutral buffered formalin for at least 24 h at 4 °C, dehydrated in ascending concentrations (70, 95 and 100%) of ethanol, cleared in xylene, and embedded in Histowax (Histo-Lab. Ltd, Gothenburg, Sweden). Five μm thick serial sections were cut, deparaffinized, rehydrated with water in descending concentrations of ethanol (100, 95 and 70%) and used for HE (hematoxylin and eosin) staining. Tissues were observed under a light microscope and a score was assigned according to the severity of the lesions, by blinded reading of the lung slides, accordingly to previously reported studies on the SRLV-induced interstitial lung disease in sheep [9-11]. Three types of lesions have been evaluated: lymphocyte infiltration i.e. presence or absence of inflammatory cells in the lung parenchyma [A], (peri)bronchiolar inflammation [B] and thickness of the alveolar septa [C]. The bronchiolar inflammation was evaluated per ten random microscopic fields using a magnification of X100 and included five categories: “0” for no lesions; “I” for mild lymphocyte inflammation around bronchiole; “II” for moderate lymphocyte infiltration around bronchiole and inside wall, epithelial bronchiolar hyperplasia; “III” for moderate to severe lymphocyte infiltration, partial destruction of bronchiolar epithelium, peribronchiolar muscle cells more visible and “IV” for destruction of bronchiolar lumen or epithelium replaced by fibrous/muscular layers with or without inflammatory cells. The thickening of the alveolar septa was evaluated according to the degree of interstitial accumulation of inflammatory cells and exudate and included four categories: “0” for no change, “I” for multifocal interstitial leukocytes without protein exudate, “II” for multifocal and intermittent confluent areas of leukocyte infiltration, with or without interstitial protein exudates and “III” for confluent areas of alveolar septal thickening with leukocytes and protein exudates.

For each horse, a lung pathology score was estimated using the [(B + C)/2] formula taking into account the severity of (peri)bronchiolar inflammation [B] and thickness of the alveolar septa [C] as previously reported [9-11]. This score was used to sort the horses according to the severity of the observed lung pathology where 0 to 0.4 means lesions, 0.5 to 1.4 defined mild lesions, 1.5 to 2.4 defined moderate lesions and >2.4 defines severe lesions.

### Expression of αSMA and p26 EIAV capsid (CA) evaluated by immunofluorescence on lung tissues

Four μm thick sections from paraffin embedded lungs were cut and placed on superfrost glass slides. All sections were dewaxed in 3 sequential 10-min xylene washes and rehydrated through 100%, 90% and 75% ethanol baths. Tissues were rinsed with tap water and PBS for 30 min and blocked with 5% normal horse serum, 1% bovine serum albumin in PBS for 30 min at room temperature. The mouse anti-αSMA (alpha Smooth Muscle Actin) (Sigma) and the mouse anti-EIAV core antigen p26 (clone EIAV 12E8.1 MAB10206, Millipore) antibodies were respectively diluted at 1/500 and 1/50 in PBS. After overnight incubation at 4 °C, tissue sections were rinsed twice with PBS and incubated for 30 min, in the dark, with 1/500 dilution of goat anti-mouse IgG Dylight488 (Eurobio) or goat anti-mouse IgG Dylight 594 (Eurobio). Tissue sections were rapidly rinsed twice with PBS and incubated with DAPI (Fluka) for 10 min at room temperature in the dark. Sections were rinsed twice with PBS and covered with fluoromount before microscopic examination.

### Western blot detection of p26

Splenic tissues from an non-infected and a naturally EIAV-infected French mare were lysed in 0.5 M Tris pH 8.0, 10% glycerol, 150 mM NaCl, 1% Triton X-100, 5 mM EDTA, 1 mM Na_3_VO_4_, 1 mM PMSF, 10 mg/mL leupeptin, 10 mg/mL, homogenized with the “Fastprep system device” (Qbiogene) and incubated for 30 min on ice. Forty μg of proteins were separated on a 15% SDS PAGE and transferred onto a 0.2-mM nitrocellulose membrane (Biorad). The membranes were pre-incubated with TSBT (25 mM Tris pH 7.6; 0.15 M NaCl; 0.05% Tween 20) containing 5% non-fat dry milk for 1 h at room temperature. After three washes in TSBT, the membrane was incubated for 1 h at room temperature with a 1/1000 dilution of the anti-p26 antibody, washed in TBST and incubated for an hour at room temperature with a 1/30 000 dilution of the anti-mouse IgG (whole molecule)-peroxidase antibody (Sigma). The immunoreactive bands were detected using the “Supersignal West Pico” reagent (Perbio).

## Results

### Lesions consistent with interstitial lung disease are present in EIAV^POS^ horses

**Figure 1:**
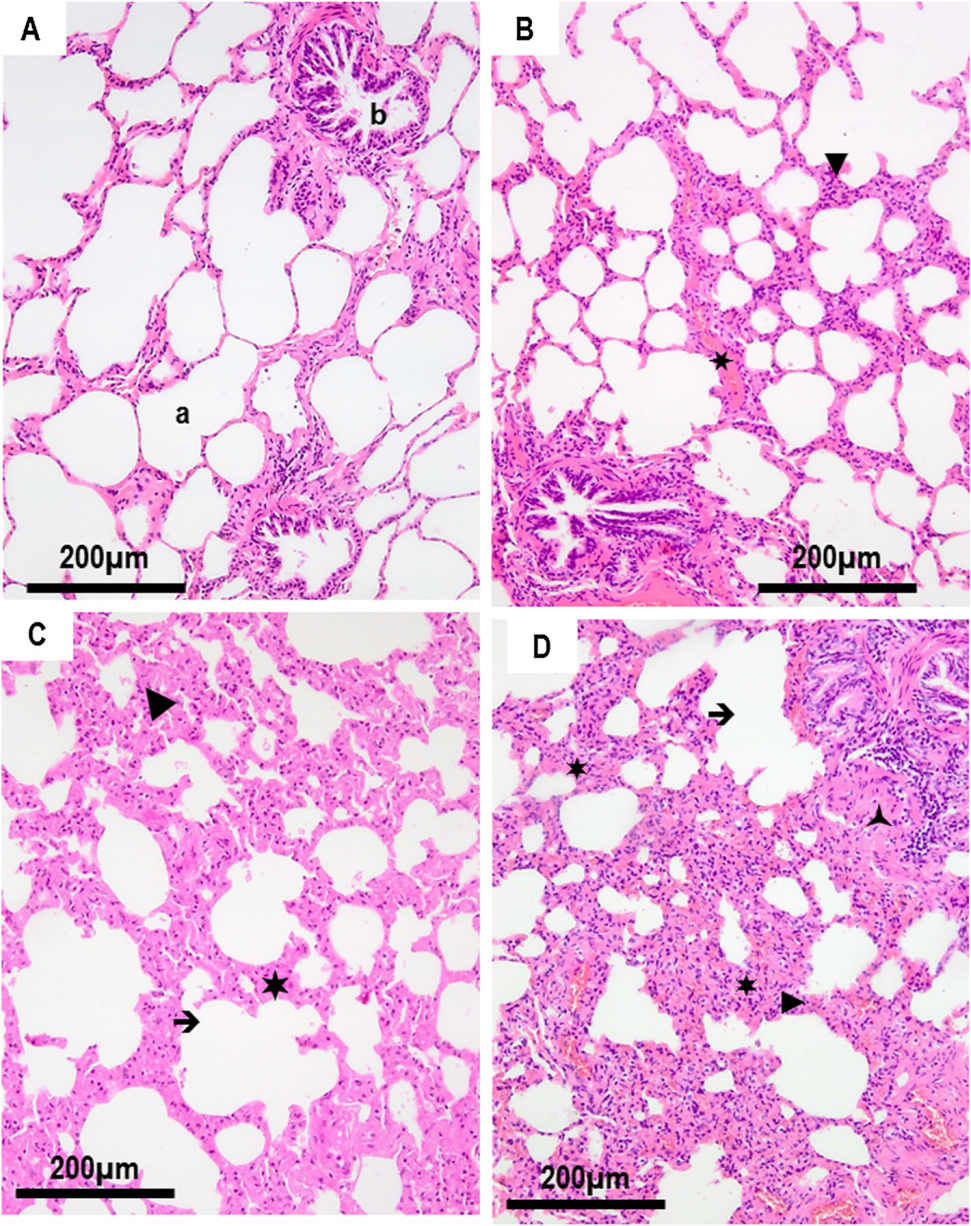
**Grading of the histological lesions from lung sections. A**. Absence of histological lesion in the lung parenchyma of an EIAV^NEG^ horse (#91-rom). **B**. Mild lesions of broncho-interstitial pneumonia in an EIAV^POS^ horse (#1-rom) revealed peribronchiolar and interstitial lymphocytic accumulation with mild thickening of the interlobular septa. **C**. Moderate lesions in EIAV^POS^ horse (#78-fr) with accumulation of lymphocytes within the septa and moderate thickening of the alveolar walls. **D**. Severe lesions of interstitial pneumonia in EIAV^POS^ horse (# 2-rom) with peribronchiolar lymphocyte infiltration, partial destruction of bronchiolar epithelium, peribronchiolar accumulation of smooth muscle cells and thickening and partial destruction of the alveolar septal leukocyte exudate. (►) Interstitial lymphocyte accumulation; (**✶**) thickening of the interlobular septa; (➔) destruction of alveolar walls; () peribronchiolar accumulation of smooth muscle cells; a: alveoli; b: bronchiole.

In contrast to the EIAV^NEG^ horses (Figure 1A), 52% of the EIAV^POS^ horses displayed a mild inflammation around the bronchioles (Figure 1B), 21.5% had moderate inflammation with inflammatory cells inside the alveolar wall and areas of epithelial bronchiolar hyperplasia (Figure 1C), and 6.3% had moderate to severe inflammation, with destruction of the bronchiolar epithelium and accumulation of smooth muscle cells (Figure 1D). Severe inflammation was present only in one horse (#16-rom) with absence of bronchial epithelium, replaced by fibromuscular layers with dispersed inflammatory cells. Among the EIAV^NEG^ horses only 3 of the 15 animals (21.4%) presented with mild inflammatory changes in the (peri)bronchiolar area. Changes in the thickness of the alveolar septa were present in the EIAV^POS^ animals. Considering this group, over 40% presented with multiple foci of mainly mononuclear cells in the septa and 26.6% displayed multifocal and intermittent confluent areas of leukocyte infiltration, without interstitial protein exudate. Among these EIAV^POS^ horses, 30% had no change regarding the thickness of septa. Except for one horse showing limited thickness and mononuclear cell infiltration into the septa, lesions were absent in the EIAV^NEG^ control group.

**Figure 2:**
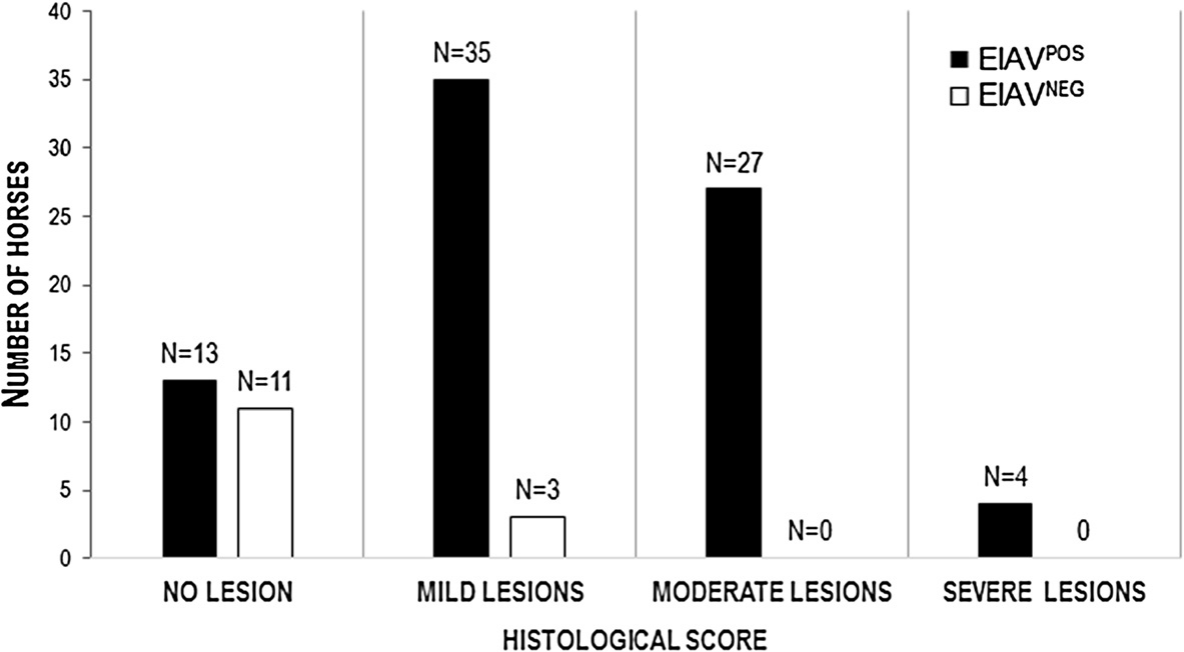
**Distribution of interstitial lung lesions in horses according to their EIAV status.** The histological scores are reported for the EIAV^POS^ and EIAV^NEG^ populations. This score was used to sort the horses according to the severity of the observed lung pathology where 0 to 0.4 means no lesions, 0.5 to 1.4 mild lesions, 1.5 to 2.4 moderate lesions and > 2.4 severe lesions. N: number of horses.

Taking together, pulmonary lesions characterized by lymphocyte infiltration, (peri)bronchiolar inflammation and thickness of the septa were present in most EIAV^POS^ horses (Table 1, Figure 1). Only mild lesions of bronchiolar or peribronchiolar inflammation and limited thickness of the alveolar septa have been observed in the EIAV^NEG^ animals. After the blind observation of all the 93 lung sections and the determination of the histological score defined by [(bronchiolar/peribronchiolar inflammation + thickness of the alveolar septa)/2], 83% of the EIAV^POS^ horses developed mild to severe lung lesions defined by a score above 0.4. In contrast, only 21% of animals from the EIAV^NEG^ group had mild lesions with a score under 1.5, with no animal with moderate or severe lesions (Table 1, Figure 2).

**Table 1:** Grading of the pulmonary lesions.

**Type of lesion**		**Number of cases**	
		**EIAV**^**pos**^	**EIAV**^**neg**^
**[A]** Lymphocyte infiltration	Absence:	**14**	11
	Presence:	**65**	3
	Grade:		
**[B]** (Peri)bronchiolar inflammation	0	**15**	11
	I	**41**	3
	II	**17**	0
	III	**5**	0
	IV	**1**	0
**[C]** Thickness of the septa	0	**24**	13
	I	**34**	1
	II	**21**	0
	III	**0**	0
	Histological score * [(B + C)/2]		
No lesions	0 (0–0.4)	**13**	11
Mild lesions	I (0.5-1.4)	**35**	3
Moderate lesions	II (1.5-2.4)	**27**	0
Severe lesions	III (>2.4)	**4**	0
	Mean score:	**1.09**	0.17

Pulmonary lesions have been blindly observed and graded according to their severity from EIAV-positive (EIAV^POS^) and EIAVnegative (EIAV^NEG^) horses. [A] described the presence of infiltrating lymphocytes. [B] described the type and the severity of (peri) bronchiolar inflammation; [C] described the thickness of the septa and its severity; * total histological score was calculated using the [(B + C)/2] formula. The histological score allows the ranking of the pulmonary disease where 0 to 0.4 means no lesions, 0.5-1.4 mild lesions, 1.5-2.4 moderate lesions and >2.4 severe lesions.

**Figure 3:**
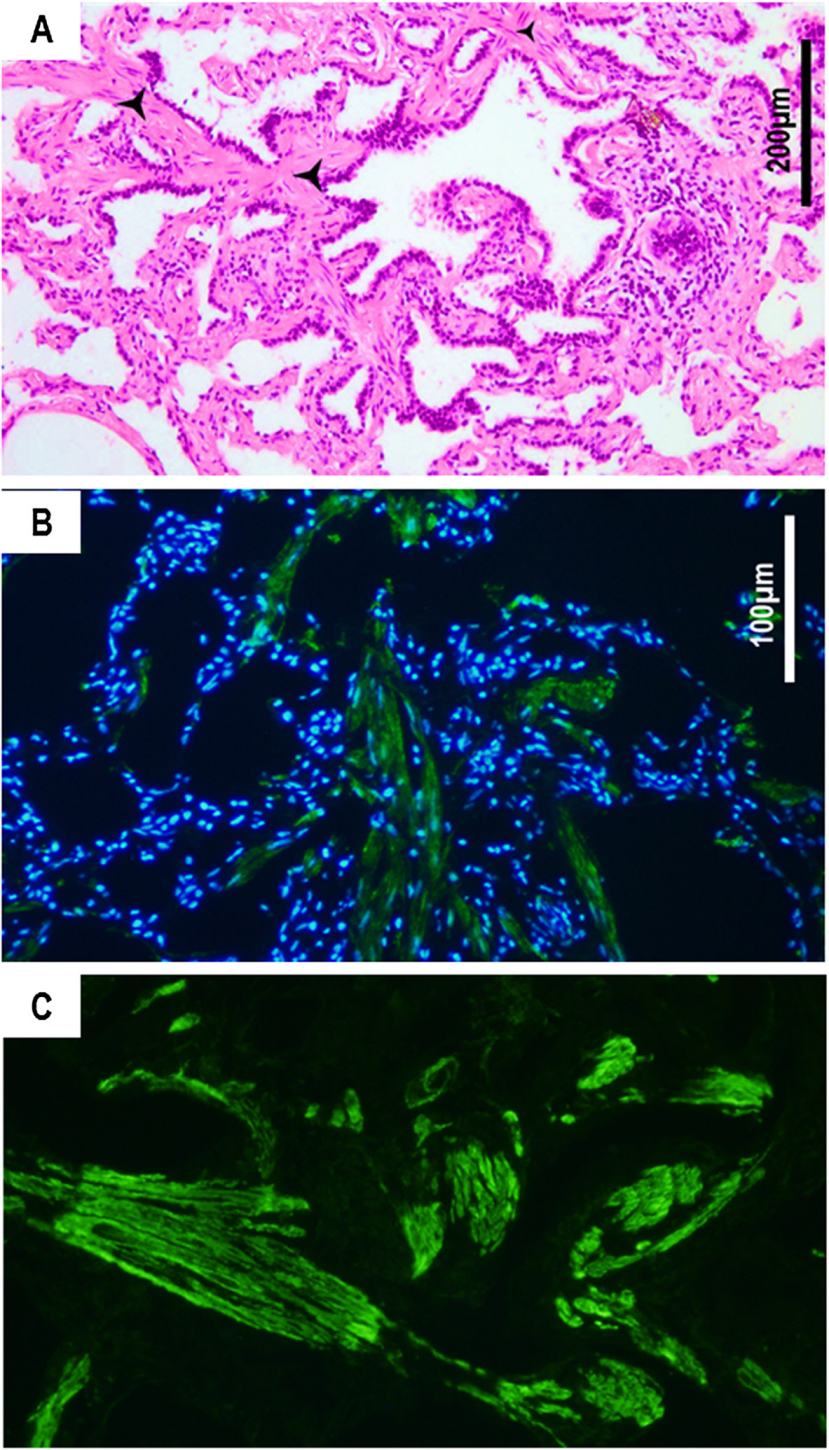
**Smooth muscle cell accumulation in the lung parenchyma (horse #16-rom). A**. Smooth muscle cells accumulate into the lung parenchyma () and disrupt the bronchiolar structure. Hematoxylin-eosin staining on paraffin-embedded sections. **B**. Immunostaining of smooth muscle cells using an anti-αSMA antibody shows the accumulation of fusiform cells (in green) within the lung parenchyma. **C**. Higher magnification shows organized actin filaments within the infiltrating cells. Nucleus are stained in blue with DAPI.

Smooth-muscle cells accumulation within the lung parenchyma has been observed in 8 EIAV^POS^-horses (10.13%) (Figure 3A). In these animals, the inflammation and destruction of the bronchiolar architecture were accompanied by smooth muscle hyperplasia or proliferation centrifugally starting from a bronchiole, accompanied by bronchiolar metaplasia of alveoli. Immunostaining with anti-αSMA antibodies evidenced large cells with organized reticulated actin- fibers (Figures 3B and 3C).

### The EIAV p26 capsid protein is expressed in the equine pulmonary cells

In order to associate the observed lesions with the expression of the EIAV viral protein, immunostainings for the p26 capsid protein using a monoclonal anti-p26 antibody have been performed on lung sections from EIAV^NEG^ and EIAV^POS^ horses. Specificity of the anti-p26 antibody has been validated by Western blot analysis on splenic protein extracts from EIAV^POS^ French horses with the detection of both the p26 CA and the PR55^gag^ precursor (data not shown).

**Figure 4:**
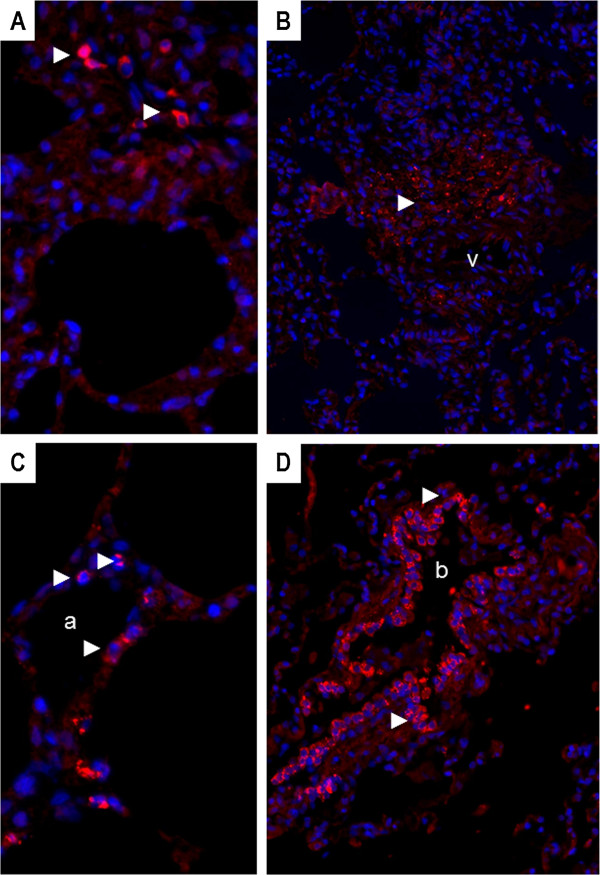
**Expression of EIAV p26 capsid in lung sections of EIAV**^**POS **^**horses. A**. Capsid expression in macrophages present in the interstitial lung tissue from an EIAV^POS^ horse (#5-rom); original magnification X400; **B**. Capsid expression in mononuclear inflammatory cells infiltrating the lung parenchyma around a blood vessel (v); horse #2-rom; original magnification X200; **C**. Capsid expression in epithelial cells of the alveolar walls, compatible with AECII and AECI; EIAV^POS^ horse #5-rom; original magnification X400; **D**. Capsid expression in bronchiolar epithelial cells or club cells (Clara); EIAV^POS^ horse #23-rom; original magnification X200; a: alveoli; b: bronchiole. Red: p26 expression (►); blue: nucleus stained with DAPI.

The p26 capsid was expressed in the infiltrated mononuclear cells within the alveolar septa (Figure 4A) and into endothelial cells of the blood vessels (Figure 4B). Interestingly, EIAV p26 was also expressed in cells consistent by their morphology and localization into the lung parenchyma with alveolar and bronchiolar epithelial cells such as type I and type II alveolar epithelial cells (AECI and AECII) in the alveoli (Figure 4C) and club cells (Clara) in the bronchiole (Figure 4D). These epithelial cells expressed p26 capsid in their cytoplasm, with a dotted pattern corresponding to local accumulation of protein (Figure 4C). Despite several attempts, phenotypic characterization of AECII by their expression of SP-C (Surfactant Protein C) failed, due to non-specific cross reaction with the tested antibodies (not shown).

## Discussion

We explored the potential of EIAV to induce pulmonary lesions in naturally infected equids from 79 EIAV-seropositive horses collected in Romania and France. We scored three types of lesions on paraffin-embedded sections: lymphocyte infiltration, (peri)bronchiolar inflammation, and thickness of the alveolar septa and analyzed the expression of the p26 EIAV capsid protein. Compared to EIAV-negative horses, over 80% of the EIAV-positive animals displayed lesions of interstitial lung disease, with an histological score above 0.4 varying from from mild inflammation around the bronchioles, to moderate inflammation with inflammatory cells inside the walls and epithelial bronchiolar hyperplasia or severe inflammation with destruction of the bronchiolar epithelium and accumulation of smooth muscle cells within the lung parenchyma. The lesions were often localized sub-pleurally concomitantly with metaplasia of the alveolar epithelium, suggesting a chronic bronchiolitis. Interestingly, the p26 EIAV capsid was strongly expressed in mononuclear cells, endothelial cells as well as alveolar and bronchiolar epithelial cells.

Smooth muscle proliferation or myomatosis was present in over 10% of the EIAV^POS^ horses as evidenced by microscopic examination of lung parenchyma and immunostaining for the expression of α-smooth muscle actin. Lentivirus infection of smooth muscle cells may play a role in the induction of pathogenic events as reported for myomatosis in the lung of SRLV infected sheep [12,13], primary pulmonary hypertension and coronary lesions as well as smooth muscle tumors of liver or ileum in HIV infected individuals [14-16], or intimal proliferation in pulmonary artery in SIV infected monkeys [17,18]. From the analyzed sections, we did not evidenced the presence of p26 into the smooth muscle cells; the link between EIAV infection and accumulation of smooth muscle cells needs to be further investigated.

Taking all together, the observed lesions resemble the lung lesions associated with LIP (lymphoid interstitial pneumonia), a lung disease described during lentivirus-infection, and reported in child infected by HIV-1 [19], cats infected by FIV [20,21] or sheep infected by SRLV [12,22,23]. LIP has been reported as a direct consequence of HIV-1 infection in humans [19] especially in child born from seropositive mothers [24]. Pulmonary disease is the major cause of morbidity and mortality in infants and children infected HIV [25]. A direct role of HIV in the induction of the observed lung disease has been reported (reviewed in [26]). Highly active antiretroviral therapy (HAART) proved to be efficient in changing the natural history of HIV infection in child, reducing opportunistic infections as well as organ specific diseases such as HIV-associated LIP [27,28]. This suggests a direct role of the lentivirus in the induction of the interstitial lung disease. In SRLV-infected sheep, the severity of the lung lesions has been correlated with the virus load [29].

We observed EIAV capsid expression in mononuclear cells as well as in endothelial cells. *In vivo*, equine infectious anemia virus (EIAV) replicates in macrophage-rich tissues [6,30,31] and monocyte maturation controls EIAV expression [32]. Infection of endothelial cells has been reported in horses experimentally-infected with cell-adapted EIAV strains during the acute phase [7]. Our results suggest that EIAV-expression in endothelial cells is maintained throughout the infection, in long term infected animals even in absence of clinical signs. Interestingly enough we evidenced EIAV-capsid expression into epithelial cells consistent with club cells in the bronchiole and type I and II alveolar epithelial cells in the alveoli. We failed to phenotypically characterize these cells for their expression of specific markers such as SP-C (surfactant protein C) or CC10 (Club cell 10 kDa) as we reported in sheep [33], due to a high background of unspecific staining (data not shown). Interestingly, unidentified-cells harboring EIAV have been reported in the alveolar walls of experimentally infected horses [34]. Except the detection of HIV-1 p24 capsid in bronchiololalveolar epithelium [35], little is known about the interaction between the lentiviruses and these specialized epithelial cells. Interestingly club cells and type II alveolar epithelial cells are the main targets of JSRV (Jaagsiekte Sheep Retrovirus), an oncogenic β-retrovirus responsible for the development of a naturally occurring pulmonary adenocarcinoma in small ruminants [33,36]. Chronic infection of alveolar and/or bronchiolar cells is likely to play a role in the maintenance of a chronic inflammation as it has been suggested for interstitial lung disease related to other lentiviral infection such as HIV. Although highly speculative, an aerosol transmission occurring from EIAV-infected pulmonary cells, as reported for SRLV [37], needs to be further estimated.

In conclusion, we described an interstitial lung disease in EIAV-infected horses associated with capsid-expression in macrophages, endothelial as well as in mature epithelial cells of the distal lung. The detection of infected pulmonary cells and of lesions consistent with lentivirus-induced interstitial lung disease opens the hypothesis of a virus transmission through aerosolized particles as reported for other retroviruses, such as SRLV or JSRV.

## Competing interests

The authors declare that they have no competing interests.

## Authors’ contributions

PB collected most tissues, carried out the histopathological analysis and the immunostainings, drafted the manuscript; MN carried out the immunostaining and tissue analysis; JLC participated to the result analysis and to the writing; CC participated to the tissues collection and to the histopathological analysis, FA carried out the fluorescent microscopy observations and helped to draft the manuscript, CD carried out the immunostaining and Western blot analysis; JFM participated to the design, to the interpretation of the results and their analysis and to the final version of the manuscript; CL conceived the study, elaborated its design, coordinated the work and wrote the manuscript. All authors read and approved the final manuscript.
